# Unlocking Efficiency in Radio-Frequency Heating: Eigenfrequency Analysis for Resonance Identification and Propagation Enhancement in Nigerian Tar Sands

**DOI:** 10.1021/acsomega.3c08484

**Published:** 2024-01-03

**Authors:** Adamu A. Adamu, Prashant. S. Jadhawar, Lateef Akanji, Sumeet S. Aphale

**Affiliations:** School of Engineering, Fraser Noble Building, University of Aberdeen, King’s College, Aberdeen AB24 3UE, U.K.

## Abstract

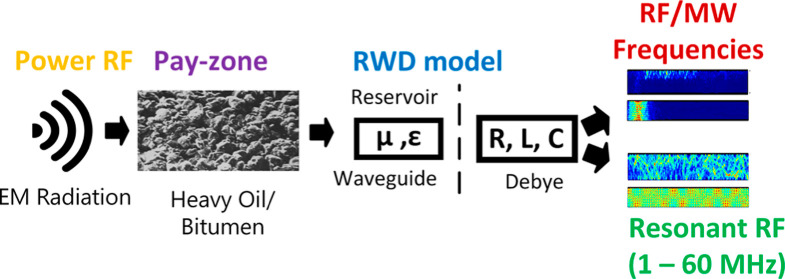

Nigerian bituminous tar sands are among the world’s largest deposits of bitumen and heavy oil. They are estimated to contain 38–40 billion barrels of heavy oil and bitumen, spanning approximately 120 km in length and 4–6 km in breadth. With global commitments to net zero emissions and various energy transition plans, improvements in the recovery methods for heavy oil and bitumen are being sought. To address this, renewable energy electrothermal enhanced oil recovery is considered an eco-friendly alternative. In our study, we introduce a novel Reservoir-Waveguide-Debye model. This model explores the enhancement of penetration for radio-frequency electromagnetic (EM) waves, which can be generated from renewable energy sources. These waves facilitate the viscosity reduction of heavy oil and bitumen. Through a comprehensive 2D numerical simulation employing the bulk properties of bituminous tar sands, we assess the propagation of EM fields within porous media. We utilize the industrial heating radio-frequency bandwidth of 1–60 MHz to conduct frequency domain investigations. Our analysis delves into propagation modes using eigenfrequency analysis, pinpointing the EM resonance of the tar sands. Furthermore, we investigate the impact of mesh refinement on the EM eigenfrequencies of porous media at both the microscale (400 μm) and macroscale (100 m in radial distance). Our results demonstrate the occurrence of resonance phenomena at complex eigenfrequencies around 27.12 and 54.24 MHz in both the microscale and macroscale models of the bituminous sands. This breakthrough research offers promising insights into harnessing renewable energy-driven EM waves for efficient thermal recovery processes in the Nigerian bituminous tar sands, thus fostering sustainable and eco-friendly energy solutions.

## 1 Introduction

Recent global developments have intensified the focus on bolstering global energy security. With the pressing concerns of climate change and the urgent need for eco-friendly approaches to energy resource extraction, the demand for efficient technologies has never been more critical. Bituminous oil sands, found abundantly in regions like Africa, the Middle East, the Americas, and Eastern Siberia,^1,2^ present a promising alternative. Globally, crude oil resources total approximately 9–11 trillion barrels (bbls), with heavy oil and bitumen accounting for over 60% of this reserve.^3^ In Nigeria, significant deposits of heavy oil and bitumen are located in vast tar sands along the South-West region.^4−6^ These sands span approximately 120 km in length and 4–6 km in width, harboring an estimated 38–42 billion barrels of oil and bitumen.^7^ Notably, these resources exhibit a low API gravity (less than 20°) and high viscosity under original reservoir pressure and temperature conditions. The key to their recovery lies in employing thermal enhanced oil recovery (EOR) methods to reduce viscosity.^8,9^ Consequently, thermal recovery of Nigerian tar sands has been considered,^10−12^ while the economic feasibility of a steam-based recovery method was investigated for the recovery of heavy oil and bitumen.^13^

Efforts to enhance the sustainability of oil production processes have led to the integration of renewable energies, such as using them for electricity generation^14^ or steam generation in thermal recovery processes.^15−17^ Additionally, researchers have explored innovative techniques, including the use of ultrasonic waves (UW) in porous media for heavy oil measurements and recovery.^18−22^ Electromagnetic (EM) energy, particularly at radio frequency (RF) and microwave (MW) frequencies, has been investigated for reservoir property measurements and thermal EOR methods.^23−27^

The EM heating (EMH) method involves the radiation of EM waves from high-power antennas into oil reservoirs, where polar molecules align with the varying polarity of the EM fields, facilitating the heating of heavy oil formations and shale rocks.^27,28^ Despite its potential, the current exploration of RF-EMH faces challenges, primarily limited penetration in thin pay-zone reservoirs. Researchers have attempted to address this limitation by adjusting input power and radiation frequency, and proposing directional radiation techniques.^29−32^ To enhance the effectiveness of EMH, a critical investigation of the mitigating factors is imperative. First, the design of RF heating regimes should consider formation stratigraphy, rheology, and electrochemistry. Second, consideration of suitable well completion methods and RF antenna designs, as well as impedance spectroscopy, is necessary. While open-hole barefoot well completion enhances EM propagation, it exposes antennas to sand accumulation and chemical contamination.^33,34^ Efforts to improve EM wave penetration have been made by combining acoustic stress with MW frequencies.^35,36^ However, these studies often focused on heat generation rather than wave propagation, leaving a gap in understanding.

In this study, we address this gap by investigating the mechanism of EM propagation in bituminous oil sands. We observed the formation’s response to identify the frequency requirements for improved EM penetration. Our approach combines background theory, including a novel Reservoir-Waveguide-Debye (RWD) method for eigenfrequency evaluation, with detailed material descriptions and computational methods. As such, this review is organized as follows: Section 2 presents the background theory describing the method and introduces the novel RWD approach. In Section 3, we detail the materials used and the computational methods employed. Section 4 discusses the modeling parameters and presents an analysis of the results obtained. Finally, Section 5 outlines our derived conclusions and provides recommendations based on the findings.

## 2 Background of Study

The history of RF EMH for EOR spans more than half a century with the design of the subsurface RF radiator. In 1956, Varian made a significant breakthrough by filing a patent outlining a method to conduct magnetic resonance (MR) at the frequency of the Earth’s magnetic field (MF). This innovation aimed to detect groundwater.^37^ Soon after, Ritchey patented a radiator capable of transmitting EM waves, leading to heating through the dielectric polarization of polar molecules.^38^ However, despite these pioneering efforts, the application of EMH for EOR is primarily focused on improving recovery rates, with limited attention given to the overall process design.

The challenges faced in the industrial application of this method were largely due to concerns regarding process efficiency and feasibility. Recognizing these hurdles, we were motivated to investigate EM wave propagation, specifically in heavy oil formations. Our research aims to address these challenges and pave the way for more efficient and feasible applications of EMH in EOR.

### 2.1 Industrial Heating RF Bandwidth

Drawing upon extensive research across various applications, it has been established that effective frequencies for industrial heating typically fall within the range of 1–60 MHz, with variable power outputs.^39,40^ Consequently, defining the frequency bandwidth is crucial for studies on effective RF-EMH for EOR. To provide a standardized framework, we have adopted the range of 1–60 MHz as the industrial heating RF bandwidth (IHRFB). The response of heavy oil-porous media to incident EM waves is inherently frequency-dependent and can be evaluated through measurable EM properties.^41^ This defined frequency range serves as the foundation for our investigations, allowing us to explore the interactions between EM waves and heavy oil formation in the pursuit of enhancing EOR processes. The frequency-dependent conductive, capacitive, and inductive properties can be denoted by complex magnetic susceptibility (μ^’^), complex conductivity (σ*), and complex permittivity (ε*), respectively. The complex permittivity is given as
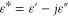
1with
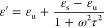
2and
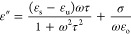
3where ε_u_ represents the relative permittivity of the upper frequency limit, ε_s_ represents the static permittivity, τ is the polarization relaxation time, σ is the electrical conductivity, ω, defined as 2π*f* is the angular frequency, while ε_o_ is the free space permittivity defined as 8.85 × 10^–12^ F/m. The permittivity has been evaluated under 60 kHz to 4 MHz.^42^ The relationship between the complex permittivity and conductivity is given as
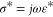
4

The frequency-dependent conductivity σ_f_ given in (S/m) was computed from the measured complex relative permittivity values in terms of the loss tangent defined by tan (δ) as
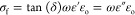
5

The response to incident MF, magnetic permeability, can be expressed in terms of mass magnetic susceptibility χ_o_ as
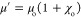
6where μ_o_ = 4π × 10^–7^ H/m. The heat generation due to instantaneous Poynting’s vector due to the EM fields is described by Poynting’s theorem as
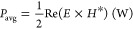
7where *E* and *H* represent the EF and MF vectors. This has been expressed in terms of penetration radius *r* from the injecting source as
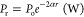
8where α is the absorption coefficient, and the subscripts “τ” and “o” represent radius and origin, respectively. Then, the derived absorbed power per unit volume is evaluated as
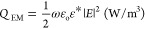
9

In the pursuit of enhancing thermal recovery through EMH, researchers have delved into the realm of solvent injection.^43−45^ This innovative approach involves injecting ferromagnetic solvents into the reservoir, which, in turn, alters the magnetic properties of the formation, affecting the bulk magnetic susceptibility (χ_o_). This parameter holds significance in determining liquid saturation in porous media.^46^ The technique has been applied in scenarios involving moderately inhomogeneous fields. For instance, a nuclear magnetic resonance (NMR) instrument known as NMR-MOUSE was employed to profile porous sedimentary rocks saturated with a combination of oil and water.^47,48^ The primary objective was to assess both the relaxation behavior of fluids and the effective application of diffusion editing methods, even in the presence of severe MF inhomogeneity.^49^

The EM response of the rock sample can be comprehensively explained using the Debye equation, a phenomenon supported by the observation of multiple relaxations within the measured frequency range.^50^ This nuanced understanding of the interplay among EM waves, magnetic properties, and solvent injections not only expands our theoretical knowledge but also offers practical insights, potentially revolutionizing the way we approach EOR.

### 2.2 Resonance Electromagnetic Radiation

In this study, resonance EM radiation (REMR) is introduced as a concept to elucidate the propagation of matching frequencies of EM waves between radiators and absorbing media. To leverage REMR for viscosity reduction in heavy oil reservoirs, it becomes imperative to identify the EM resonant frequencies specific to the formation under consideration. While methods to determine acoustic eigenfrequencies have been reported in the literature,^51^ the exploration of EM eigenfrequencies in oil formations for EOR remains an underexplored area of research. This gap in knowledge underscores the significance of our current study. By delving into the uncharted territory of determining EM eigenfrequencies in oil formations, we aim to provide valuable insights that could potentially revolutionize the field of EOR. The identification of these frequencies holds the key to unlocking the full potential of REMR for efficient and targeted viscosity reduction in heavy oil reservoirs, thereby significantly contributing to the advancement of EOR techniques.

### 2.3 Novelty: Conceptual Reservoir-Waveguide-Debye Model

**Figure 1:**
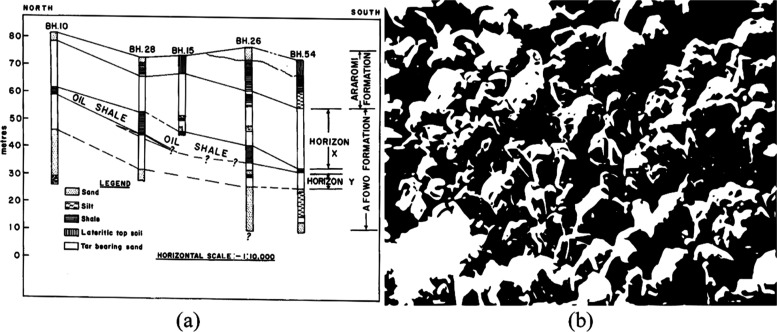
(a) Cross-sectional stratigraphic schematic of Nigerian tar sands. (b) Bitmap representation of an electron micrograph showing the high porosity of Nigerian tar sands. “Adapted from E. I. Enu, “Textural characteristics of the Nigerian tar sands”, *Sediment. Geol.,* vol. 44, no. 1–2, pp. *65–81,***1985,** with permission from Elsevier”.

**Figure 2:**
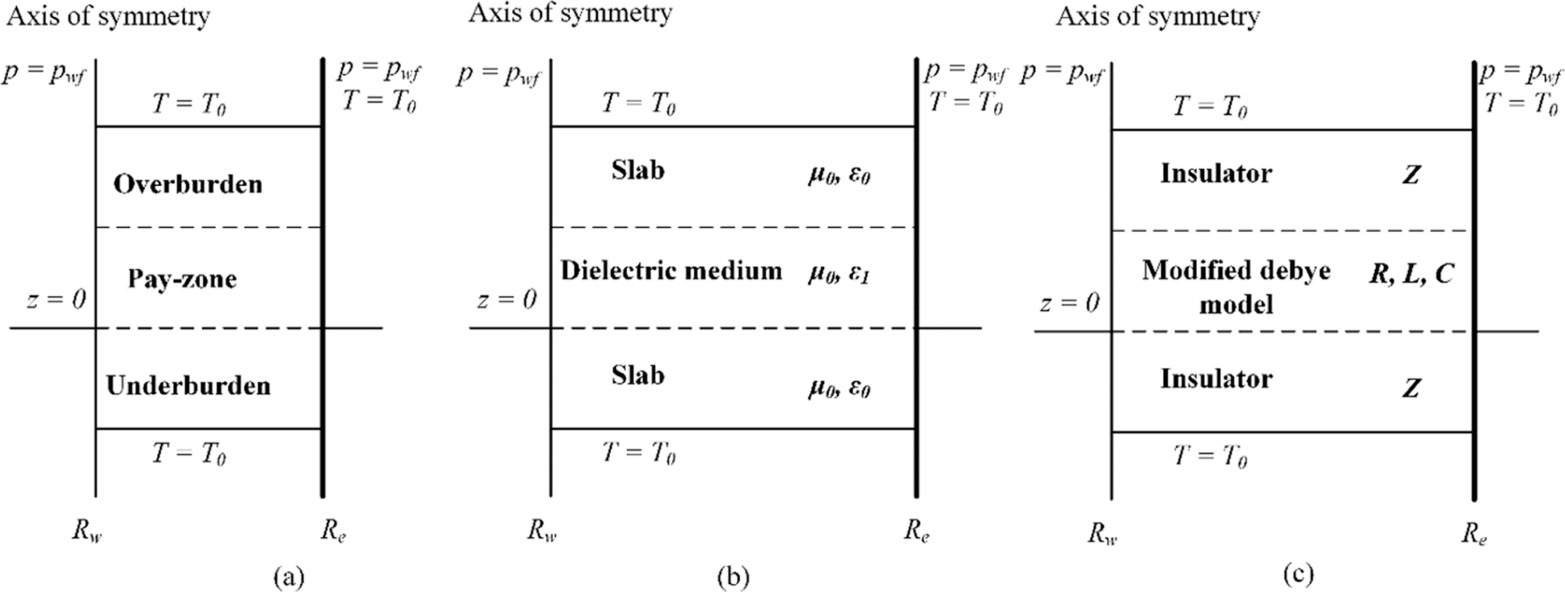
Schematic of a homogeneous RWD model showing correlation within the three respective domains. (a) Reservoir domain with preliminary boundary conditions, (b) waveguide domain with preliminary boundary conditions, and (c) Debye domain with preliminary boundary conditions.

In our study, the exploration of EM resonance is crucial due to its potential to enhance energy transfer and subsequent heat generation within formations. To accurately gauge the effectiveness of this phenomenon, it is necessary to evaluate the distance over which the EM waves penetrate the reservoir. Therefore, we interpret the reservoir formation at a macroscale, considering radial distances ranging from 1 to 100 m. In this context, our analysis adopts a cross-sectional perspective of the multilayered reservoir, revealing a structural analogy resembling a slab waveguide.^52^ This configuration, based on the topography as seen in Figure 1a, is characterized by a dielectric medium (pay zone) sandwiched between layers of slab at the top and bottom.^53^ To comprehend the absorption of EM waves within the formation, we employ the Debye model, providing an electrical representation. Here, the formation’s resistivity, complex permittivity, and bulk magnetic susceptibility correspond to their respective impedance counterparts: resistance (*R*), capacitance (*C*), and inductance (*L*). Consequently, these elements form three representative domains within our conceptual RWD model, as illustrated in Figure 2.

**Figure 3:**
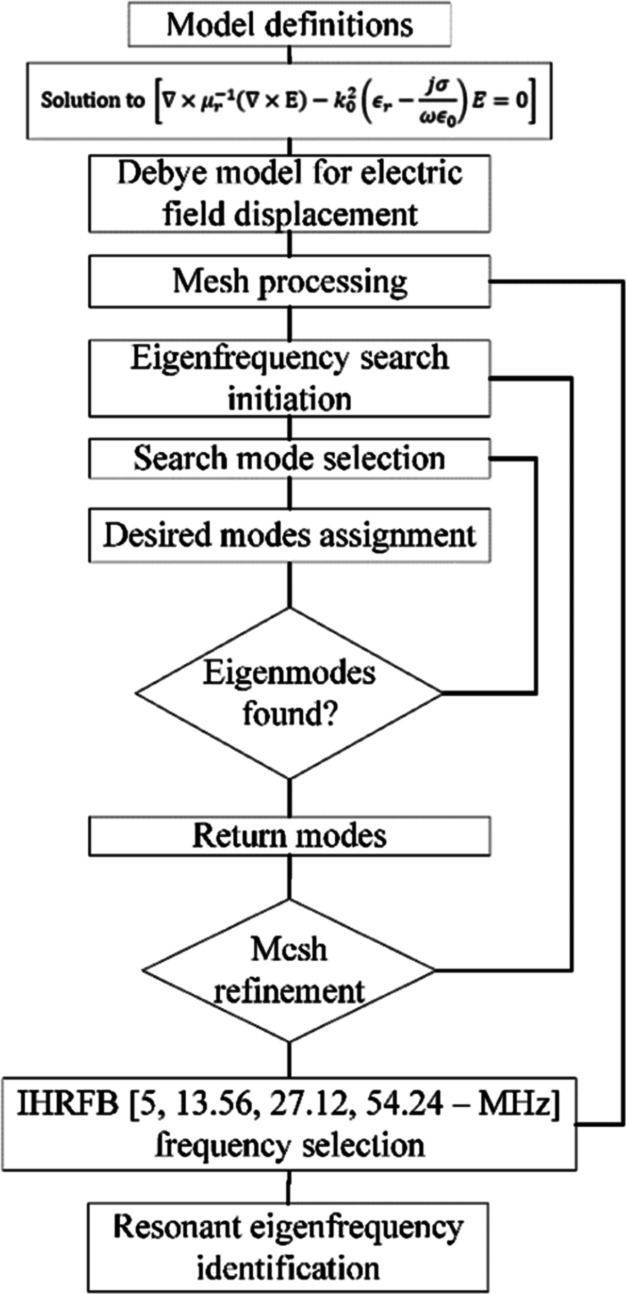
Solution workflow for the determination of eigenfrequencies within the IHRFB.

**Figure 4:**
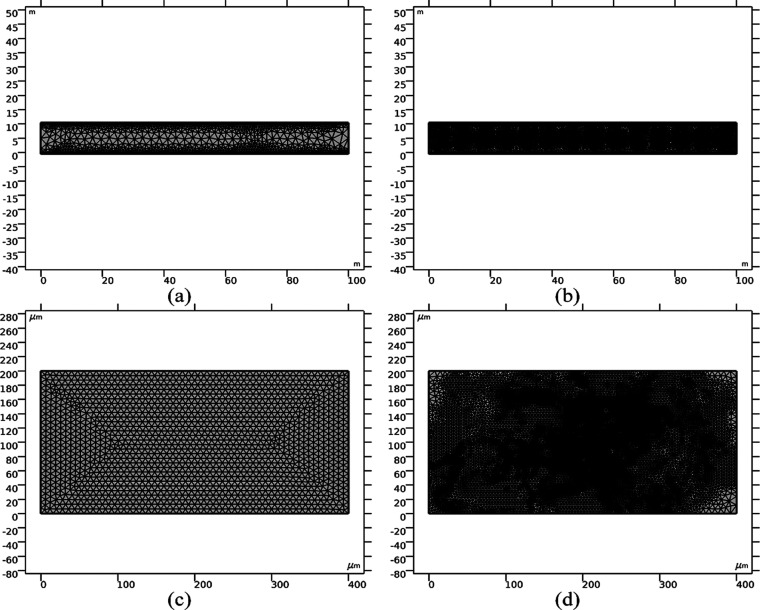
Model showing (a) the first and (b) third level of mesh refinement of the macroscale model outlining the formation porous matrix, while (c) the first and (d) third level of mesh refinement of the microscale model composition of the tar sands.

This comprehensive model serves as the foundation for our exploration, enabling a detailed understanding of the EM resonance within the reservoir. By integrating theoretical concepts with practical applications, we aim to unravel the intricacies of wave penetration distances and pave the way for the more effective utilization of EM resonance in enhancing energy transfer and heat generation within oil formations (Figures 3 and 4).

The wave propagation within bituminous sands, interpreted as a dielectric medium, can be accurately described through the equations governing a planar dielectric waveguide. Theoretically, this waveguide can support two distinct modes of propagation: the transverse electric (TE) mode and the transverse magnetic (TM) mode. By combining these modes, complete EM fields within the medium can be obtained. Assuming symmetry, the fields remain independent of the *z*-coordinate.^31^ The waveguide interpretation of porous media stems from our interest in understanding field propagation, which is a crucial factor that influences the losses incurred within the EMH regime. Although the model represents an assumption of reservoir homogeneity, the heterogeneous nature of the formation can be approximated by the equations of the multilayered (inhomogeneous) dielectric waveguide. Acknowledging the macroscale nature of porous media, the waveguide representation is valid, as the wavelength of the EM waves within IHRFB is much larger than the physical dimensions of molecules. The nonuniformity of the media implies spatially varying permittivity, permeability, and conductivity.^53^ This is described in Appendix eqs A1–A3. This mathematical description of guided wave propagation forms the basis of our analysis, allowing us to delve deep into the intricacies of wave behavior within bituminous sands. Through these fundamental equations, we gain valuable insights into the EM properties of the medium, paving the way for more precise and efficient EMH applications in the context of oil reservoirs. The distribution of the guided power transmitted by the source (antenna) is given by
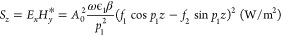
10where *z* is the height of the pay zone (m), β is the propagation constant (rad/m), and *A*_0_ is an unknown coefficient, which depends on source excitation. The time-average power flow is given as
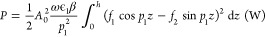
11

In single-mode propagation, the presence of a complex dielectric constant within the material implies that waves attenuate through the medium. This can be represented by
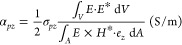
12where σ_*pz*_ is the conductivity of the pay zone, *A* is the unit surface area (m^2^), *V* is the unit volume of the medium (m^3^), σ_*pz*_ = ωϵ^’’^,ϵ = ϵ^’^ – *j*ϵ^’’^, , while *E* and *H* are the EF and MF of the guided mode. With dielectric loss in the pay zone, then
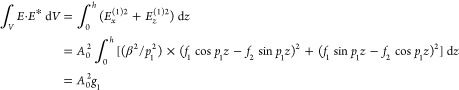
13whereas
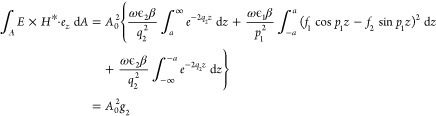
14effectively
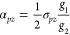
15

The TE surface wave modes may be treated as TM modes. For the TE case, the nonzero field components are (*H*_*z*_, *E*_*x*_, and *E*_*y*_). The appropriate expression for the pay zone region is
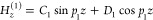
16
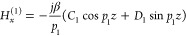
17
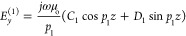
18and
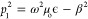
19with *C*_1_ and *D*_1_ being arbitrary constants. From the perspective of wave absorption, we characterize the dielectric pay zone (oil sand) from the perspective of impedance spectroscopy.

### 2.4 Determination of EM Eigenfrequency

Frequency-dependent variations in both the magnitude and phase of fluid and solid velocities are well-documented phenomena, offering insights into the porosity and permeability of the medium. Pan and Horne conducted pivotal research on the resonant behavior of saturated porous media, assuming periodic media structures and applying frequencies ranging from 0.1 to 10 Hz.^51^ Their findings revealed significantly larger magnitudes of the solid rigidity modulus, fluid flow rate, and solid vibration at resonance, indicating the potential for enhanced oil production through vibration at resonance frequencies. Analytical solutions for natural frequencies are limited to simple geometries, leading to widespread adoption of the finite element method (FEM). Displacement-based fully compatible FEM provides upper-bound solutions for eigenfrequencies.^54^ When considering EM resonance in porous media, it is essential to account for both MF and electric field (EF) contributions. Research exploring MF contributions in both water-wet and oil-wet samples has shown increased recoveries in both cases.^55^

Regarding EF, resulting flows manifest as electro-osmotic flows due to the polarization of the polar pore matrix and the presence of an electrically conductive fluid forming an electrical double layer (EDL).^56^ These detailed observations shed light on the intricate interplay among EM resonance, porous media properties, and fluid dynamics, providing valuable insights for the optimization of EOR techniques.

## 3 Materials and Methods

Inspired by the methodology outlined in^51^ and building upon Soedel’s prior analysis of resonance in dry solid porous mediums,^57^ our focus centers on a macroscale model. This model aims to evaluate eigenfrequencies within the IHRFB ranging from 1 to 60 MHz. In our study, we consider a pay zone saturated by tar sands.

To assess the frequency response of this saturated porous medium, we conduct a comprehensive analysis of eigenfrequencies, employing a steady-state approach tailored to the unique characteristics of Nigerian tar sands. We closely examine the textural properties of these sands in comparison to the well-known Athabasca oil sands.^4,58,59^ The oil-impregnated sands within the Nigerian formation exhibit a net thickness of 27 m, featuring a clay content ranging from 2 to 7% and an oil content between 8 and 16%. Given the variability in intergranular voids from one sample to another, our analysis yields a range of porosity values spanning from 24 to 35%. These pores constitute tar, water, and air, emphasizing the complex and dynamic nature of the porous medium under investigation.^4^

### 3.1 Model Design

The presence of porous media phenomena with varying lengths and time scales poses challenges to the predictive capabilities and computational efficiency of models. To address this complexity, macroscale models of pore-scale processes have been instrumental in enhancing the computation of transport phenomena within porous media.^60^ In our study, we adopt a macroscale model to investigate EM propagation and resonance identification in porous media. The electrical conductivity remains relatively constant up to approximately 1 MHz and exhibits a linear increase in log-space from 1 × 10^–3^ to 1 × 10^–1^ S/m for higher frequencies, specifically RF and MW frequencies, in correlation with the level of wetness. Simultaneously, the electrical dielectric constant decreases from around 40 to 3 at MW frequencies.^61^ Additionally, specific heat capacity values, crucial for understanding thermal behavior, have been reported.^62^ These essential properties are summarized in Table 1, providing a foundational framework for our detailed investigation into EM propagation and resonance phenomena within the porous media context.

**Table 1:** Table of Model Geometrical and Material Properties

geometrical properties					
name	expression	value	name	expression	value
model scale	macro	(m)	model scale	micro	μm
radial distance	100 [m]	100 m	radial distance	400 [μm]	4 × 10–^6^ m
height	10 [m]	10 m	height	200 [μm]	2 × 10–^6^ m

material properties			
name	value	description	ref
*f*_o_	1.356 × 10^7^ Hz	variable operational frequency (5, 13.56, 27.12, and 54.24 MHz)	
DC	30	dielectric constant (relative permittivity)	(42)
RP	0.8	relative permeability	(63)
EC	0.01 S/m	electrical conductivity	(63)
resistivity	1000 Ω·m	resistivity	(7)
SHC	0.6 kJ/(kg·K)	specific heat capacity	(64)

To validate the outcomes acquired from the macroscale eigenfrequency evaluation, we delve into a microscale model. The dimensions of this model are derived from detailed scanning electron microscopy (SEM) analysis.^4^ The intricacies of the models are further delineated through the corresponding mesh refinements, as outlined in Table 2. These models are meticulously computed utilizing COMSOL Multiphysics 5.6, employing the RF module, running on a CPU Intel64 Family 6 Model 78 Stepping 3 with 2 cores, operating on the Windows 10 operating system. This microscale exploration adds a granular level of detail to our analysis, allowing us to corroborate and augment the findings obtained at the macroscale. By bridging the gap between macro and micro perspectives, we aim to gain a comprehensive understanding of EM propagation and resonance phenomena within porous media, thereby enriching the depth of our research insights.

**Table 2:** Mesh Refinement Parameters for Macroscale Model

description	value		
refinement level	0	1	2
minimum element quality	0.7519	0.2579	0.1966
average element quality	0.9955	0.9348	0.8905
triangle	31,334	59,722	136,505
edge element	782	798	1329

### 3.2 Computational Method

We assume that the waves are generated from an isotropic source. A quadratic discretization method is applied for the solution of the EF equation. Then the solution of the wave equation is computed via
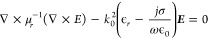
20with λ = −*j*ω + δ and ***E*** = *Ẽ*(*x,y*)*e*^–*jk*_z_*z*^, respectively. Disregarding overburden and underburden complexities, as well as intricate wave reflection phenomena, our focus narrows to the pay zone layer within the reservoir, targeted specifically by RF radiation. Utilizing the FEM to unravel eigenmodes during RF-EM wave propagation, we employed an eigenvalue solver algorithm. In our frequency domain study, the EM wave interface within the RF module becomes our operational arena. Employing an algorithm rooted in the Arnoldi Package (ARPACK), we navigate the eigenvalue problems. This solver, adapting to varying desired modes, seeks eigenvalues closest to the absolute value of shift σ_s_. To optimize results, the method computes the largest eigenvalues, with adjustments made in the desired number of modes to minimize symmetric modes and align with the mesh refinement level.^65^ Furthermore, we enhance precision through re-evaluation using adaptive meshing, refining our approach iteratively. In the absence of a macroscale stratigraphic image of the Nigerian tar sands, we resort to the SEM image depicted in Figure 1b to define the geometry of our microscale model. For the macroscale, we assume a pay zone thickness of 10 m. Leveraging the measured electrical properties of oil sands within the IHRFB, we assign values for relative permittivity, electrical conductivity, relative permeability, and bulk susceptibility. This meticulous process ensures a rigorous and accurate microscale representation of our porous media.

## 4 Results and Discussion

The models developed based on the RWD concept were computed with variable parameters of frequency with corresponding simulation times, as presented in Table 4. In our observations, pore-scale mesh refinement revealed a proportional representation of oil sands concerning porosity. The finer mesh elements intricately captured the oil-filled pore spaces, whereas the larger mesh elements delineated the solid matrix, creating a comprehensive depiction of the porous media. These mesh element specifications are detailed in Tables 2 and 3 for the macroscale and the pore scale, respectively. Our exploration targeted eigenfrequencies within the range of 1–60 MHz within the IHRFB. During this investigation, we closely scrutinized the effects of mesh quality on the number of eigenfrequencies. The generated plots vividly illustrate EF and MF penetration within the oil-filled porous media. Surface plots visually represent EF intensity, while contour lines delineate the MF distribution along the model geometry. Additionally, tangential MF arrows indicate the magnitude of the field. However, it is noteworthy that the figures depict relatively low values of the normalized surface MFs concerning the EF norm.

**Table 3:** Mesh Refinement Parameters for Microscale Model

description	value		
refinement level	0	1	2
minimum element quality	0.8832	0.45	0.3565
average element quality	0.9915	0.9632	0.9347
triangle	3150	8704	29,169
edge element	150	200	351

**Table 4:** Computational Parameters for Dual Scale Model

scale	frequency [MHz]	refinement	DoF	internal DoF	solution time (s)
macro	54.24	0	14,443	11,920	564
		1	32,627	27,448	
		2	68,821	58,444	
	27.12	0	14,443	11,920	445
		1	27,275	22,912	
		2	59,495	50,488	
	13.56	0	14,443	11,920	143
		1	27,755	23,320	
		2	62,175	52,768	
	5	0	14,443	11,920	94
		1	31,420	26,374	
		2	72,196	61,222	
micro	54.24	0	22,351	18,904	412
		1	59,123	50,320	
		2	148,102	126,490	
	27.12	0	22,351	18,904	262
		1	58,692	49,966	
		2	129,278	110,362	
	13.56	0	22,351	18,904	541
		1	61,558	52,426	
		2	116,046	99,082	
	5	0	22,351	18,904	114
		1	57,441	48,868	
		2	92,826	79,186	

**Figure 5:**
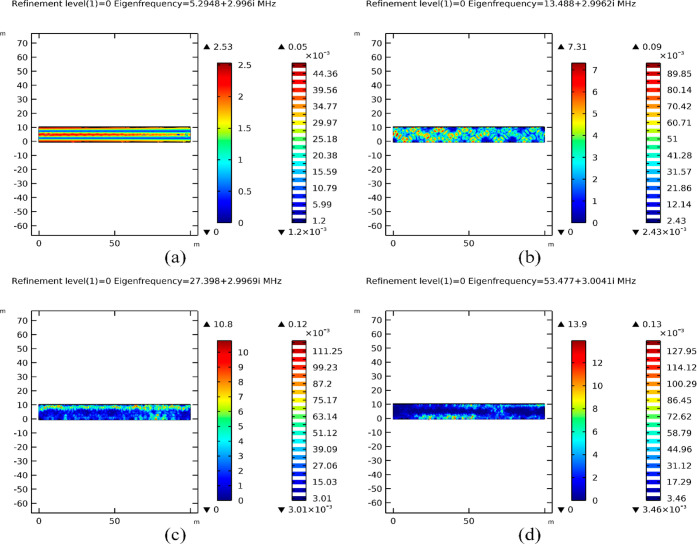
Macroscale propagation of EM fields with partial resonance at eigenfrequencies around 5–60 MHz without mesh refinement, (a) formation response at 5.2948 + 2.996*i* MHz, (b) wave propagation pattern at 13.488 + 2.9962*i* MHz, (c) formation response at 27.398 + 2.9969*i* MHz, and (d) formation response at 53.477 + 3.0041*i* MHz.

Utilizing the primary mesh (refinement level 0) properties as depicted in Figure 5, and the parameters in Table 2, we present the frequency response of the macroscale model at crucial frequencies of interest spanning from the lower bound to the upper bound of the IHRFB. In Figure 5a, the resonant response at the eigenfrequency of 5.2948 + 2.996*i* MHz is showcased. The penetration of EM waves is vividly observed throughout the cross-section of the model, indicating complete wave penetration. However, within the desiccated zones (depicted in blue), regions of poor wave energy absorption are apparent. In Figure 5b, a distinct wave propagation pattern emerges at the eigenfrequency of 13.488 + 2.9962*i* MHz. Notably, there is an increased area of desiccated zones, signifying regions where wave energy absorption is less efficient. These observations underscore the nuanced dynamics of wave propagation and absorption within the porous media, revealing critical insights for optimizing EMH applications within oil reservoirs.

Figure 5c,d illustrates the limited penetration of EM waves within the formation, resulting in low energy absorption at eigenfrequencies 27.398 + 2.9969*i* MHz and 53.477 + 3.0041*i* MHz, respectively. Following mesh refinement (refinement level 1), we meticulously compared the effects of enhanced mesh quality on eigenfrequency identification. Our observations revealed distinct modes with partial resonance closely aligned with those observed under primary mesh conditions, indicating the robustness of our findings.

**Figure 6:**
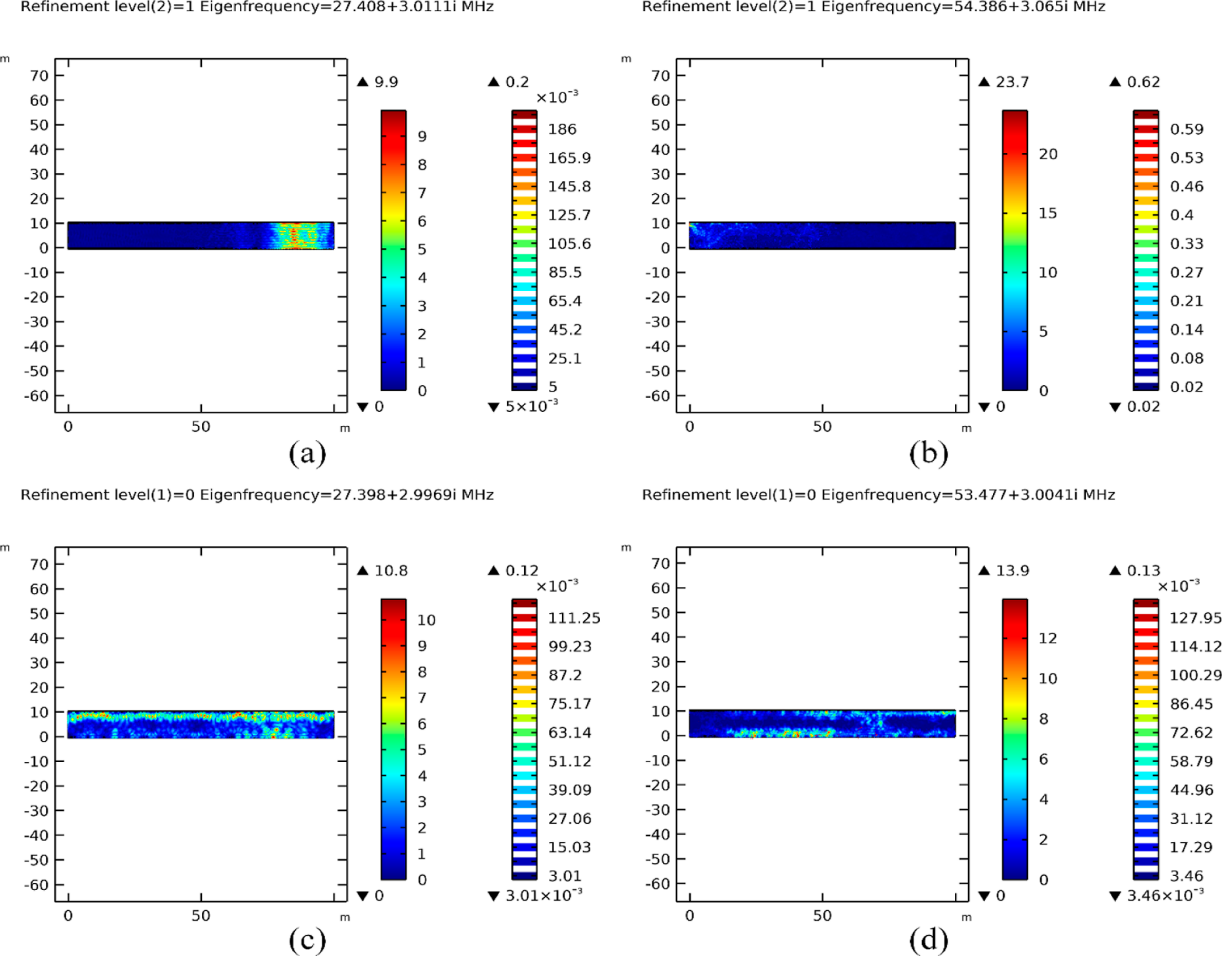
Effect of mesh refinement on eigenfrequency determination. Low penetration of EM fields at 1st level mesh refinement (a,b) between 27.12–54.24 MHz upper band eigenfrequencies with partial penetration of EM fields observed at initial mesh configuration in (c,d).

Figure 6a showcases concentrated partial resonance at 27.408 + 3.0111*i* MHz, while Figure 6c depicts distributed partial resonance at 27.398 + 2.9969*i* MHz. These results highlight the nuanced variations in resonance patterns due to enhanced mesh quality. Meanwhile, Figure 6b illustrates poor propagation at 54.386 + 3.065*i* MHz in comparison to the distributed partial resonance obtained at 53.477 + 3.0041*i* MHz, as shown in Figure 6d. These findings underscore the critical role of mesh refinement in capturing subtle nuances within the porous media’s response to EM waves, thereby providing valuable insights for refining our EMH strategies within oil reservoirs.

Distinct fully resonating eigenfrequencies, characterized by the saturation of EM field lines across the cross-section of the model, were observed within the frequency range of 27.12–54.24 MHz. During resonance, increased wave absorption leads to reduced values of the surface EF norms, a phenomenon evident in the maximum EF norm values recorded at 9.9 and 10.8 V/m in Figure 6a,c, respectively.

**Figure 7:**
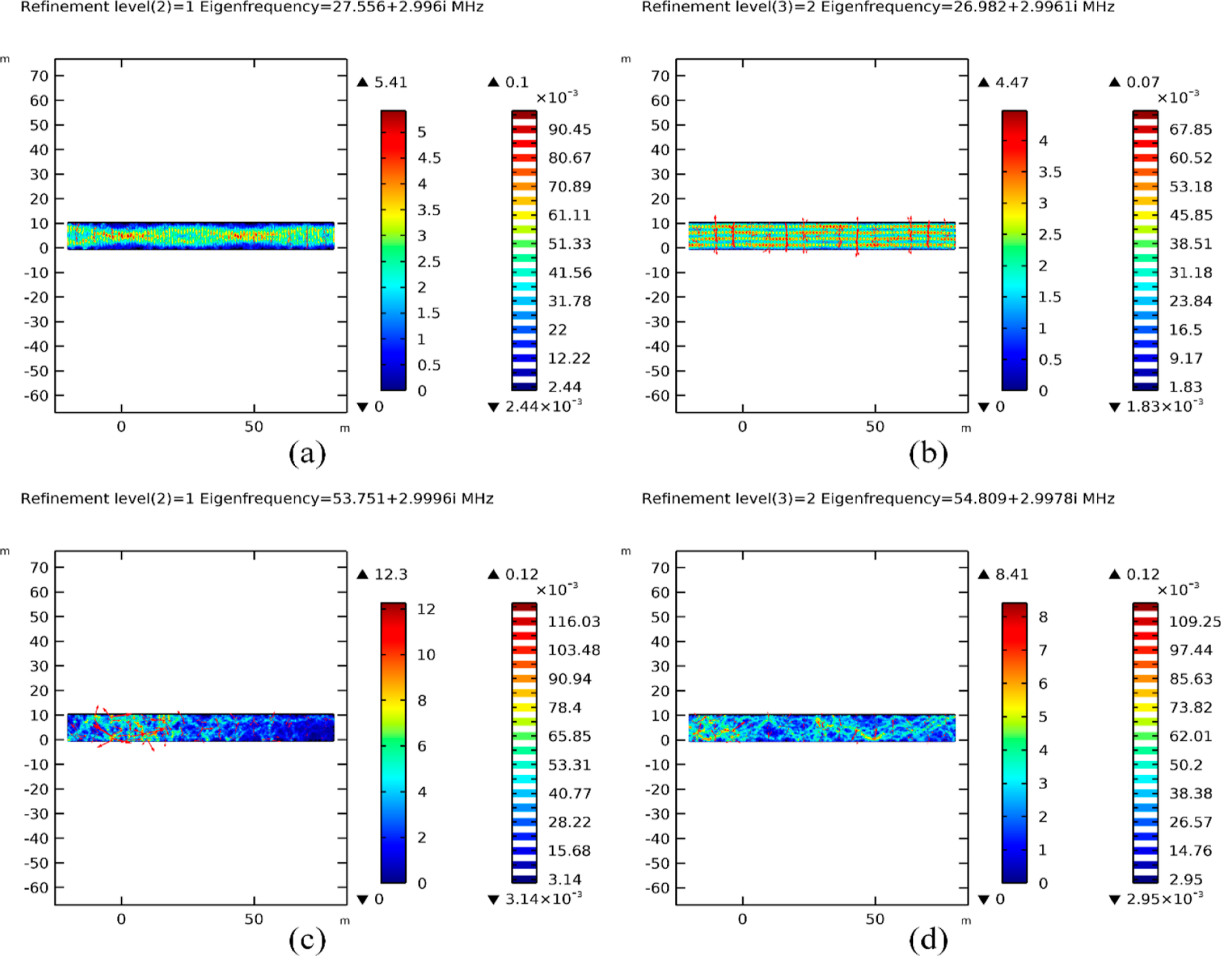
Effect of mesh refinement on resonant mode intensity at eigenfrequencies between 27.12 and 54.24 MHz, (a) resonant response at 27.556 + 2.996*i* MHz with refinement level 2, (b) resonant response at 26.982 + 2.9961*i* MHz with refinement level 3, (c) resonant response at 53.751 + 2.9996*i* MHz with refinement level 2, and (d) resonant response at 54.809 + 2.9978*i* MHz at refinement level 3.

Figure 7 provides a detailed comparison of mesh refinement around the observed resonating eigenfrequencies. Under refinement level 2, as depicted in Figure 7a, the resonance intensity at 27.556 + 2.996*i* MHz was lower than that observed at 26.982 + 2.9961*i* MHz under refinement level 3, as shown in Figure 7b. Similarly, Figure 7c illustrates a similar scenario at 53.751 + 2.9996*i* MHz under refinement level 2, with increased intensity observed at 54.809 + 2.9978*i* MHz under refinement level 3, as illustrated in Figure 7d. These findings emphasize the critical role of mesh refinement in capturing the intricate dynamics of resonance patterns, providing crucial insights into optimizing EMH strategies within oil reservoirs.

**Figure 8:**
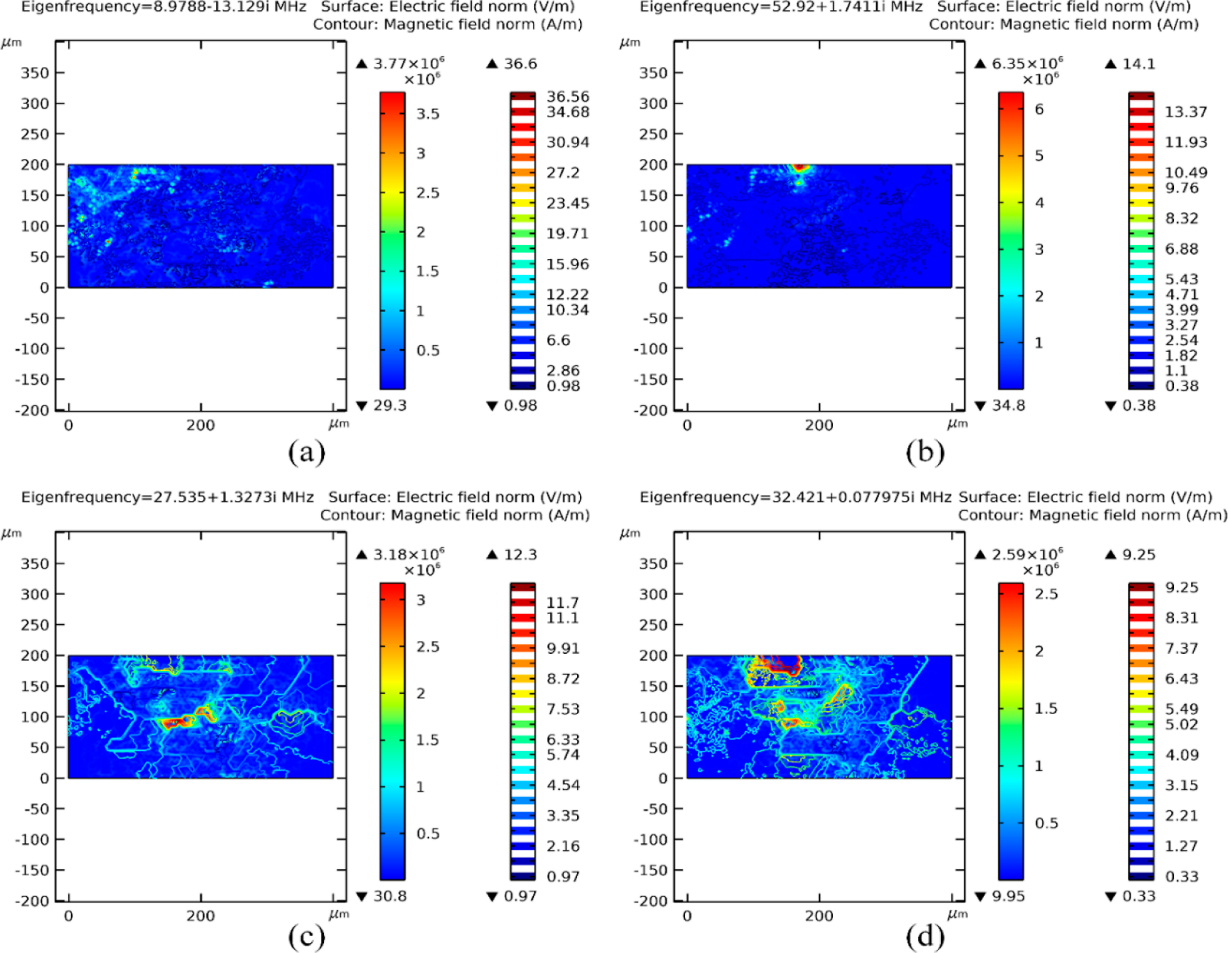
Low penetration due to poor resonance at (a) 8.9788 – 13.129*i* MHz and (b) 52.92 + 1.17411*i* MHz eigenfrequency with enhanced wave penetration due to partial resonance at (c) 27.535 + 1.3273*i* MHz and (d) 32.421 + 0.077975*i* MHz eigenfrequency.

At the micro level, resonant modes demonstrate the intricate interaction of EFs with polarized pore surfaces, marked by sharp edges, as depicted in Figure 8. Here, we observe low penetration due to poor resonance at 8.9788 – 13.129*i* MHz and 52.92 + 1.17411*i* MHz, as shown in Figure 8a,b, respectively. This interaction disperses the concentration of field lines propagated along the IHRFB. During resonance at 27.535 + 1.3273*i* MHz and 32.421 + 0.0779751*i* MHz, we observe that EF intensity is minimized due to the increased distribution of the field across the domain surface, as presented in Figure 8c,d. This phenomenon corresponds to the heightened transfer of EM energy at resonant modes, indicating a complex interplay between field concentration and energy absorption.

With the implementation of adaptive mesh refinement with mesh parameters presented in Table 3, we note a variance in the obtained eigenfrequencies, signifying increased accuracy of the solution. Notably, the revelation of imaginary components of the eigenfrequencies based on the complex electrical properties of the formation adds depth to our understanding. Like the macroscale model, we observe varying penetration at eigenfrequencies within the IHRFB at the micro level. This underscores the substantial impact of mesh quality on eigenfrequency identification, emphasizing the importance of precision in measurements for subsequent investigations of EM eigenfrequencies. These findings highlight the intricate dynamics of EM wave interactions within porous media and emphasize the need for meticulous measurement and modeling for accurate interpretations and successful application in practical scenarios.

## 5 Conclusions

The enhancement of the heating range in RFH for the enhanced recovery of heavy oil and bitumen has been a challenge for researchers. Previous efforts were focused on altering formation electrical properties by using solvents to increase conductivity. In this work, we approached the challenge from the perspective of EM wave propagation. Through a finite element frequency domain simulation, we identified the optimal frequency bandwidth for EM wave propagation within the IHRFB.

Through the introduction of the RWD model, this study sheds light on resonance phenomena within porous media based on the electrochemical properties of the Nigerian tar sands, which are comparable to the more investigated Athabasca oil sands.

With the discovery of resonant EM eigenfrequencies around 27.12 and 54.24 MHz at macroscale and confirmed at microscale, improved propagation of EM waves by tuning EM wave transmission for enhanced transfer of EM energy can be achieved. Although this work has assumed the homogeneity of the formation, heterogeneous parameters require more computational resources to evaluate. Additionally, this research forms the basis for designing and optimizing experimentation and field evaluations of enhanced thermal recovery of heavy oil and bitumen using RF EM waves. This research serves as a foundation to explore EM resonance in diverse geological settings. These explorations could lead to advanced computational models, refining our understanding of EM wave interactions at micro- and macroscales.

Moreover, this research has implications for energy exploration, potentially unlocking previously inaccessible oil reserves. Furthermore, it aligns with the industry’s focus on eco-friendly practices, reducing environmental impact. Insights from this research could lead to the development of specialized EM resonance devices, precisely targeting oil-rich zones within complex geological formations and maximizing recovery efficiency. This knowledge evolution is essential for the advancement of oil recovery technologies and geophysics.

In summary, this research not only deepens our understanding of EM resonance in porous media but also drives innovation, encourages sustainable practices, and contributes to the energy industry’s transformation. The optimized propagation of EM waves within bituminous oil sands demonstrates the potential of interdisciplinary research in shaping the future of energy exploration and environmental stewardship.

## Appendix A

### Formation Spatial Heterogeneity

To account for the spatial anisotropy of the formation, we represent the reservoir as a multilayered waveguide. Then we assume the expressions for the field components of all modes to be multiplied by the factor *e*^*–j*β*z+j*ω*t*^. Then, expressions for the tangential fields to the interface in the layers can be expressed for the pay zone as

A1

where , , *q*_1_^2^ = β^2^ – *k*_1_^2^, , and *k*_*m*_^2^ = ω^2^ μ_*m*_ϵ_*m*_.

Then, the matrix definition  is made. To simplify large matrices representing multiple layers of the structure, a compressed Yeh and Lindgren technique is applied as

A2

with

A3

for the multiple layers obtainable in a practical formation.

## Notes

Subject Classification Codes: Improved and Enhanced Oil Recovery, Oil Sands, Bitumen, Radiofrequency, and Thermal Recovery.

## Abbreviations

EM: electromagnetic
MW: microwave
EMH: electromagnetic heating
RF: radiofrequency
RF EMH: radiofrequency electromagnetic heating
RWD: Reservoir-Waveguide-Debye
MF: magnetic field
EF: electric field
EOR: enhanced oil recovery
NMR: nuclear magnetic resonance
UW: ultrasonic waves
IHRFB: industrial heating radiofrequency bandwidth
SAGD: steam-assisted gravity drainage
EC: electrical conductivity
RP: relative permeability
SHC: specific heat capacity
DC: dielectric constant

## Symbols and Notations

ε*: complex permittivity (F/m)
*j*: imaginary unit number
τ: polarization relaxation time (s)
ε_u_: relative permittivity of the upper frequency limit (dimensionless)
ε_s_: static permittivity (F/m)
σ: electrical conductivity (S/m)
ω: angular frequency (rad/s)
*f*: frequency of EM wave (MHz)
*f*_o_: variable Input frequency (MHz)
ε_o_: material permittivity (F/m)
Re: real part of complex number
σ*: complex electrical conductivity (S/m)
*P*_avg_: average absorbed power (W)
μ^’^: magnetic permeability (H/m)
χ_o_: mass magnetic susceptibility (m^3^/kg)
*E*: electric field vector (V/m)
*H*: magnetic field vector (A/m)
α: absorption coefficient (dimensionless)
β: propagation constant of the field (dimensionless)
*r*: penetration radius (m)
*S*_*z*_: transmitted power (W)
*A*: arbitrary coefficient of excitation (dimensionless)
*Q*_em_: absorbed electromagnetic power (W/m^3^)
*T*: reservoir temperature (°C)
*T*_0_: initial reservoir temperature (°C)
*z*: vertical axis of pay zone (m)
*p*: reservoir pressure (Psi)
*p*_wf_: pressure at well foot (Psi)
*R*_w_: radial axis of wellbore (m)
*R*_e_: radius of external boundary (m)
*R*: resistance (ohm)
*L*: inductance (H)
*C*: capacitance (F)
*Z*: electrical impedance (ohm)
SHC: specific heat, J/(kg °C)
